# Non-covalent Fc-Fab interactions significantly alter internal dynamics of an IgG1 antibody

**DOI:** 10.1038/s41598-022-13370-3

**Published:** 2022-06-04

**Authors:** Ramakrishnan Natesan, Neeraj J. Agrawal

**Affiliations:** Amgen Inc., Process Development, 360 Binney St, Cambridge, MA 02141 USA

**Keywords:** Computational biophysics, Biomedical engineering, Biological physics, Protein function predictions, Protein structure predictions

## Abstract

The fragment-antigen-binding arms (Fab1 and Fab2) in a canonical immunoglobulin G (IgG) molecule have identical sequences and hence are always expected to exhibit symmetric conformations and dynamics. Using long all atom molecular simulations of a human IgG1 crystal structure 1HZH, we demonstrate that the translational and rotational dynamics of Fab1 and Fab2 also strongly depend on their interactions with each other and with the fragment-crystallizable (Fc) region. We show that the Fab2 arm in the 1HZH structure is non-covalently bound to the Fc region via long-lived hydrogen bonds, involving its light chain and both heavy chains of the Fc region. These highly stable interactions stabilize non-trivial conformer states with constrained fluctuations. We observe subtle modifications in Fab1 dynamics in response to Fab2-Fc interactions that points to novel allosteric interactions between the Fab arms. These results yield novel insights into the inter- and intra-fragment motions of immunoglobulins which could help us better understand the relation between their structure and function.

## Introduction

Immunoglobulins, also known as antibodies, are secreted by B-lymphocytes and play key roles as mediators and effectors of humoral and adaptive immunity^1^. Engineered antibodies are widely used as therapeutic agents across a spectrum of human conditions, including cancers. To date, over 100 antibodies (biologics) have been approved in the US and EU and a few hundreds more are in various stages of clinical evaluations^2^. Antibody (Ab) sequence, structure, and function are intrinsically related to each other and understanding this relationship is essential for effective Ab-engineering, particularly for affinity optimization^3,4^.

An immunoglobulin G (IgG) Ab is a heterodimer constituted of 2 identical heavy chains (HC1 and HC2) and 2 identical light chains (LC1 and LC2). This polypeptide assembly is organized into a fragment-crystallizable (Fc) and two fragment-antigen-binding (Fab1 and Fab2) domains. The Fc and Fab domains are typically depicted as a **Y**-shaped structure with the Fc at the stem, Fab1 and Fab2 at the flanks. Each Fab region is covalently linked to the Fc via short linkers called the hinges. Abs are highly flexible molecules with their conformations spanning the entire range from **Y** to **T**, by virtue of which they can bind to a wide variety of antigens, differing in shape, size, and sequence^5,6^. However, crystallizing all conformer states of these highly flexible molecules remains a challenge and as a result so far only very few full-length crystal structures of human IgGs have been resolved^7,8^. IgG1 b12 (pdb id: 1HZH), the first human IgG structure to be resolved, interestingly shows asymmetric organization of the Fab^5^. A recent study by Zhang et. al. mapped Ab conformations using individual particle electron tomography and constructed 120 different Ab conformer states, most of which did not adhere to the symmetric **Y**-structure^9^.

It is commonly believed that the internal dynamics of Fab1 and Fab2 are identical, based on the assumption that all conformers from **Y** to **T** are part of a smooth continuous free energy landscape. While this is true for most conformers, non-covalent interactions between Fc and Fab can lead to more complex free energy profiles which in turn can alter the internal Ab dynamics. Particularly, the presence of Fc-Fab interaction in the human IgG1 b12 structure (pdb id: 1HZH) has been noted in multiple studies^5,6,10,11^. However, the effect of these interactions on the dynamics of the Ab has not been studied in detail.

In this article, we study the internal dynamics of the human IgG1 structure 1HZH using long all atom molecular dynamics simulations that allows us to monitor the individual and collective motions of all atoms. Our analysis shows non-covalent Fc-Fab interactions severely constrain the translational and rotational degrees of freedom of the Ab which in turn stabilizes ground states that have constrained fluctuations.

## Results

**Figure 1:**
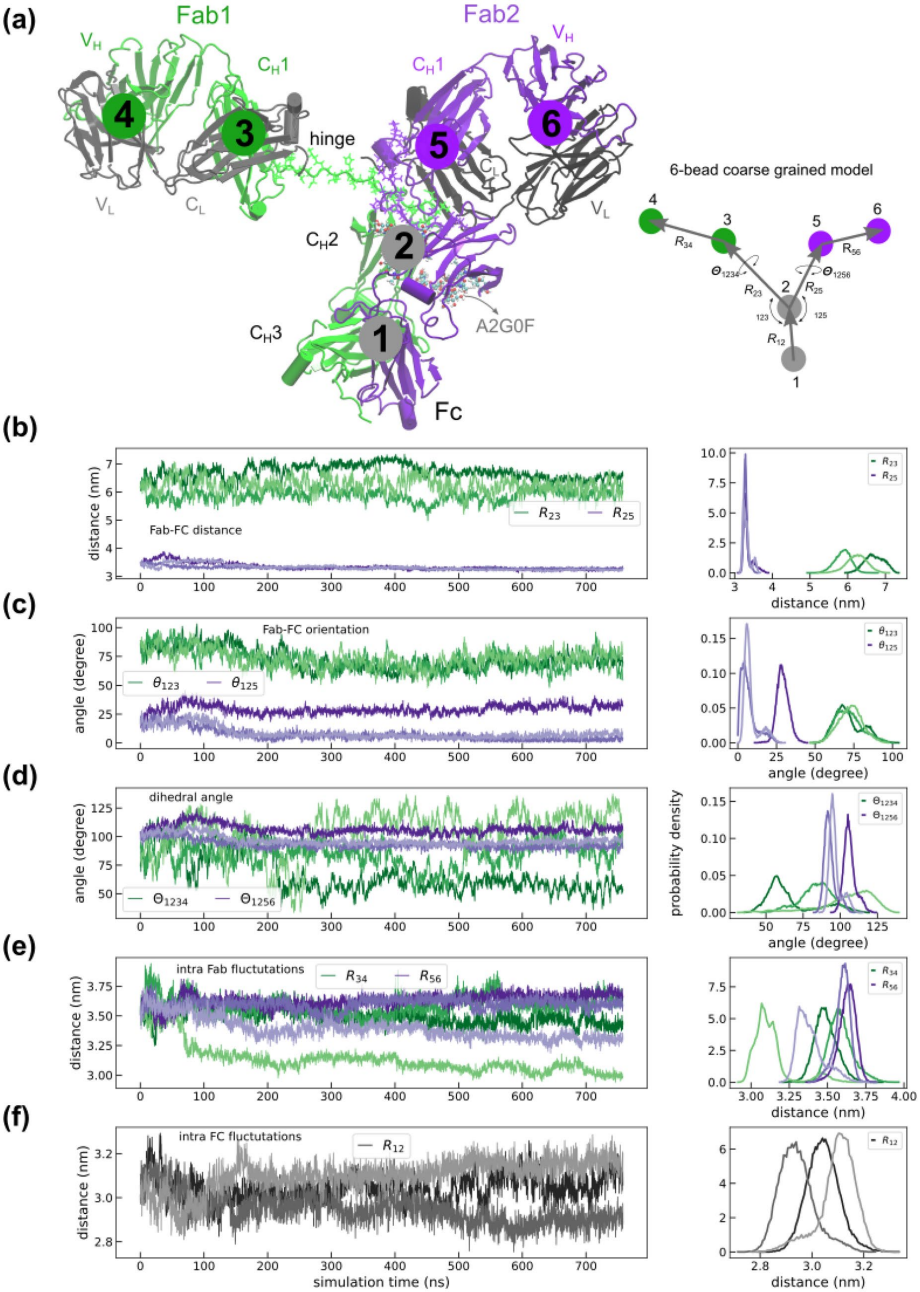
(**a**) A snapshot of N-glycosylated 1HZH structure showing Fc, Fab1 and Fab2 fragment alongside their constant and variable domains. The hinges are represented as lines and the N-glycosylated A2G0F glycans attached to residues HC1:N297 and HC2:N297 are show as beads. The Ab is coarse grained into six-distinct regions associated with the following domains. **1**: Fc C_H_3, **2**: Fc C_H_2, **3**: Fab1 C_H_1 + C_L_, **4**: Fab1 V_H_ + V_L_, **5**: Fab2 C_H_ + C_L_, **6**: Fab2 V_H_ + V_L_. The illustration on the right shows the various distance and orientation measures computed from the simulations. ${R}_{ij}$ is the distance between particles $i$ and $j$, ${\theta }_{ijk}$ is the angle subtended by particles $i,j$ and $k$, and ${\Theta }_{ijkl}$ is the dihedral angles between the planes formed by particles $i,j, k$ and $l$. The statistics of these measures are quantified in (**b-f**) where the left and right panels display the time series and probability distributions, respectively. Shown are (**b**) Fab-Fc displacements ${R}_{23}$ and ${R}_{25}$, (**c**) Fab-Fc orientation ${\theta }_{123}$ and ${\theta }_{125}$, (**d**) Fc-Fab dihedral angles ${\Theta }_{1234}$ and ${\Theta }_{1256}$, (**e**) intra Fab displacements ${R}_{34}$ and ${R}_{56}$, and (**f**) intra-Fc displacement ${R}_{12}$. Data from three independent replicates are shown using lines of similar colors.

We performed three independent 750 ns NPT simulations (E1-E3) of an IgG1 molecule (based on the 1HZH crystal structure with N-glycosylated A2G0F) at 300 K, as described in the “Materials and methods” section. A snapshot of the protein at around 1 ns is shown in Fig. 1(a), wherein the hinge regions and glycans are differently represented for clarity. We analyzed the trajectories using the six-bead framework shown in Fig. 1(a). In our model, the beads represent the center of masses of their associated Ab domain. Beads **1** and **2** correspond to the C_H_3 and C_H_2 region of the of Fc, respectively. Similarly, the C_H_1 + C_L_ regions in Fab1 and Fab2 were mapped to beads **3** and **5** while the corresponding V_L_ + V_H_ regions were mapped to beads **4** and **6**, as shown in Fig. 1(a). In our analysis, the positions of all beads were computed for every frame of the trajectory. The statistics of inter- and intra-fragment fluctuations for the three replicates are displayed in Fig. 1(b)-(f).

### Fab2 exhibits constrained distance and orientational dynamics compared to Fab1

We computed ${R}_{23}$ and ${R}_{25}$, the average distances of the Fab1 and Fab2 constant regions (beads 3 and 5) with respect to the Fc C_H_2 domain (bead 2), respectively. The time series for ${R}_{23}$ and ${R}_{25}$ is shown in Fig. 1(b) where we find the equilibrium separation of ${R}_{23}$ to be in the range of 5–7 nm compared to ${R}_{25}$ that is tightly constrained to a very small range around 3.5 nm. The latter also displays tighter fluctuations in all three replicates (E1-E3) pointing to non-identical ground states and dynamics for each Fab arm. Our analysis indicates that Fab2 is more constrained than Fab1. This marked difference is also reflected in the Fab arms orientational fluctuations ${\theta }_{123}$ and ${\theta }_{125}$ displayed in Fig. 1(c). Our analysis shows Fab1 orientational dynamics (${\theta }_{123})$ is identical across all replicates and follows a broad Gaussian distribution. On the other hand, Fab2 orientational fluctuations (${\theta }_{125})$ are severely constrained and show inter-replicate variations. The distribution profile of ${\theta }_{125}$ for each replicate follows a narrow Gaussian distribution. Similarly, the Fab dihedral angle fluctuations also reveal constrained dihedral motion in Fab2 compared to Fab1, see Fig. 1(e). However, there is no asymmetry in the intra-Fab distance fluctuations shown in Fig. 1(e) which is identical to intra Fc fluctuations shown in Fig. 1(f). A similar analysis by Marti et. al. where they studied the dynamics of 1HZH tethered to a silica surface using molecular dynamics simulations did not show the pronounced asymmetry observed in our simulations^10^. Identical asymmetric Fab dynamics was also observed in simulations of glycosylated-1HZH in explicit solvent conditions, thus confirming the validity of our simulations. Results from 500 ns explicit solvent studies of 1HZH are displayed in Supplementary Figure S1. We also observed non-identical Fab dynamics in simulations of non-glycosylated 1HZH thus ruling out any role for the glycan groups in constraining Fab2 degrees of freedom. Results from 350 ns simulations of non-glycosylated 1HZH is shown in Supplementary Figure S2. Other measures such as the elbow angle^12,13^ did not show a significant difference between the two Fab arms, see Supplementary Figure S3.

### 1HZH essential dynamics is dominated by Fab1 motion

**Figure 2:**
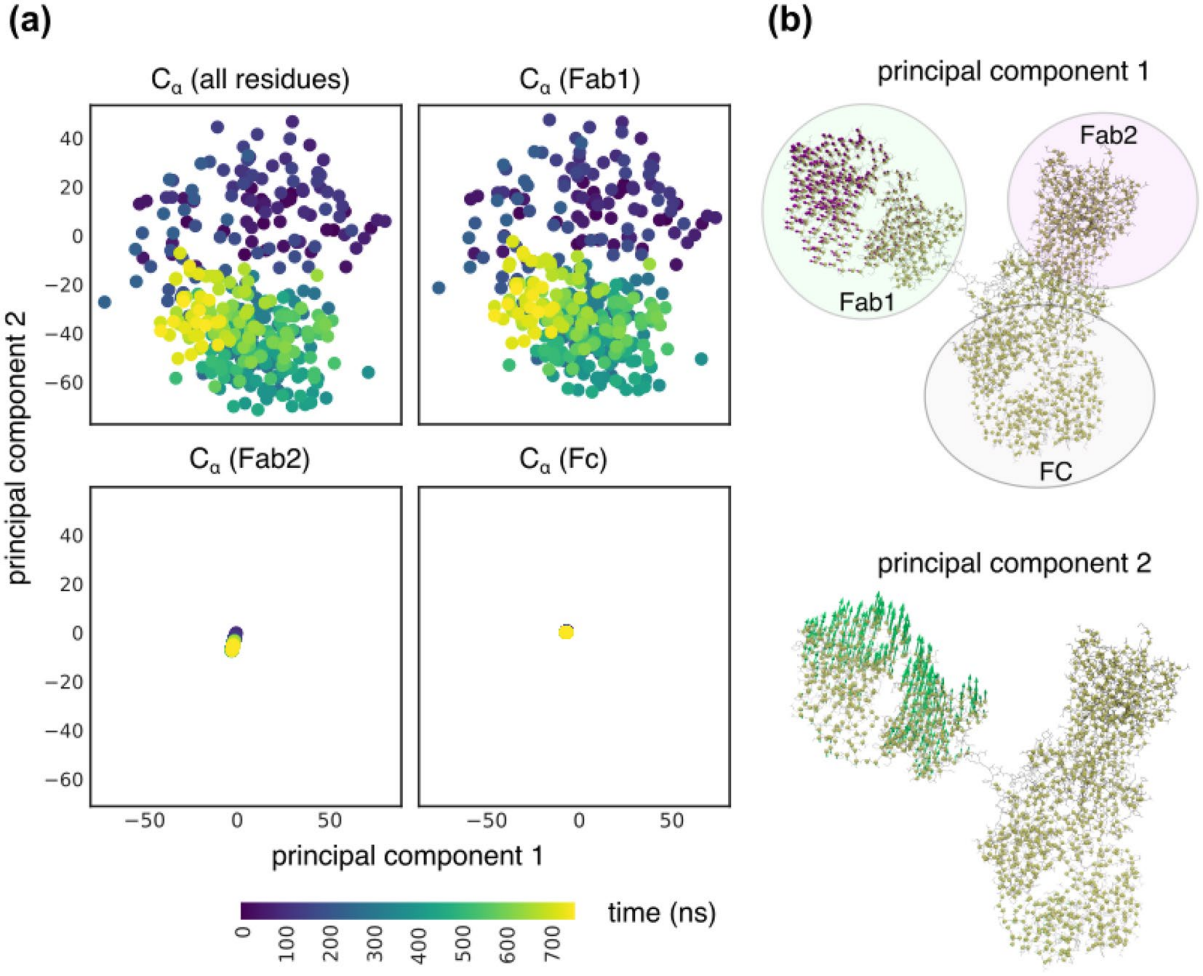
(**a**) Projections of protein ${C}_{\alpha }$ positions along principal components 1 and 2 shows that the essential dynamics of the protein is entirely governed by Fab1 dynamics. Contributions from Fab2 and Fc along the first two principal directions are negligible. (**b**) Snapshots of 1HZH showing the contributions of each ${C}_{\alpha }$ atom to principal component 1 (top) and principal component 2 (bottom). The size of the arrows is directly proportional to the contributions and the backbone alpha carbons are displayed as spheres.

Analysis of essential dynamics is a powerful approach to identify residues that dominate the motion of the protein^14^. We studied the essential dynamics of 1HZH by performing a principal component analysis of the ${\mathrm{C}}_{\mathrm{\alpha }}$ atom positions, as described in the “Materials and methods”. Results from our analysis is displayed in Fig. 2. Projections of all ${\mathrm{C}}_{\mathrm{\alpha }}$ atoms along principal components 1 and 2 closely follow the dynamics of the ${\mathrm{C}}_{\mathrm{\alpha }}$ atoms in the Fab1 arm, as shown in Fig. 2a. Similar projections of ${\mathrm{C}}_{\mathrm{\alpha }}$ atoms in the Fab2 arm and Fc showed negligible contributions along the first two principal vectors. In fact, we observed negligible contributions from Fab2 and Fc for the first four principal vectors as shown in Supplementary Figure S4. The dominant motion of Fab1 is clearly illustrated in Fig. 2b where the contribution from each ${\mathrm{C}}_{\mathrm{\alpha }}$ atom to principal components 1 and 2 is displayed by its corresponding vector. It can be clearly seen that the dominant principal vectors are associated only with ${\mathrm{C}}_{\mathrm{\alpha }}$ atoms in the Fab1 arm, which as described in Fig. 1 explores a larger conformational space compared to Fab2 and Fc. These results strongly support our observation that Fab2 in 1HZH exhibits a highly constrained dynamics compared to Fab1.

### Persistent Fc-Fab2 interactions govern the differential dynamics of Fab2

**Figure 3:**
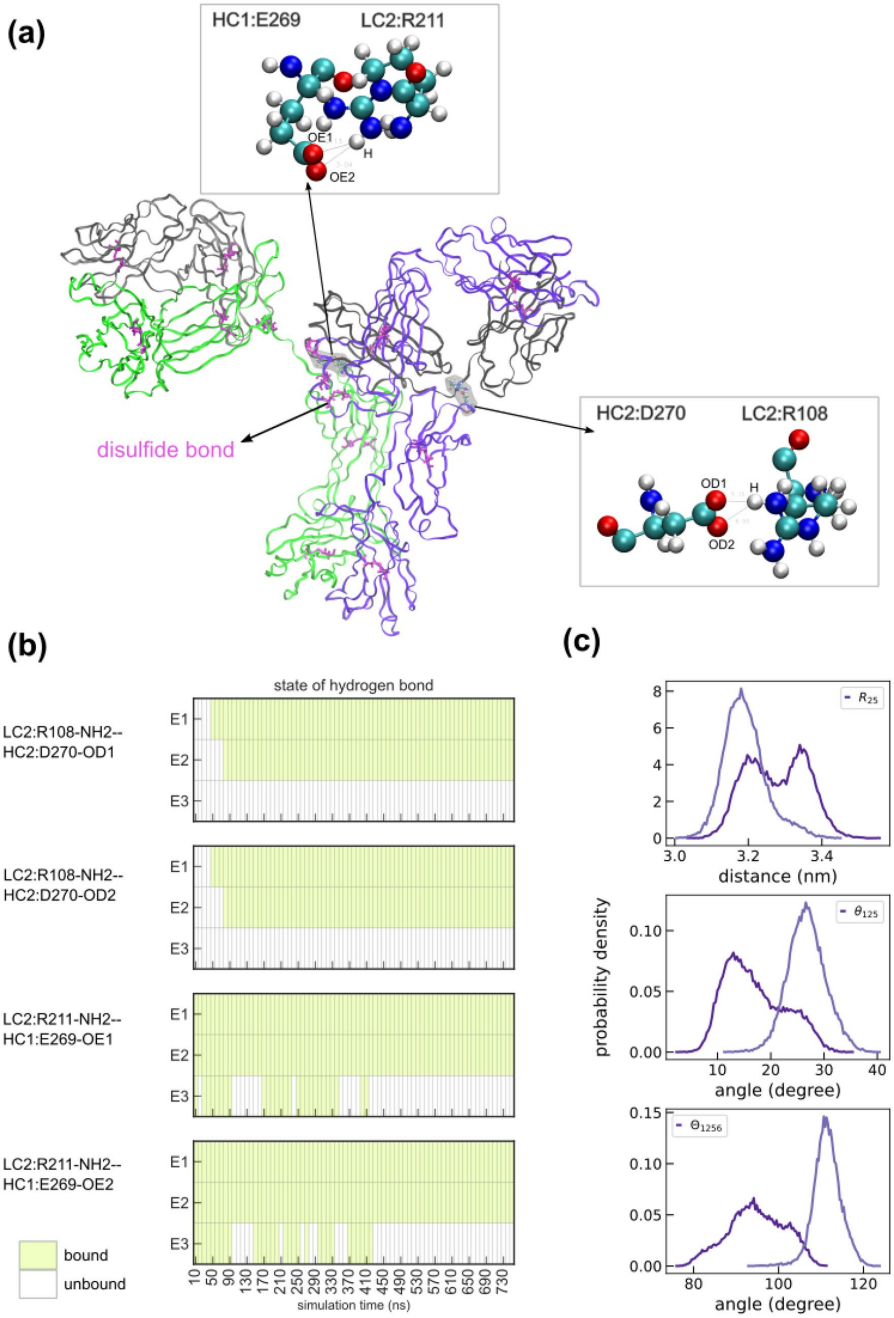
(**a**) Cartoon representation of N-glycosylated 1HZH with the 16 disulfide bonds represented using the bond representation. Beads enclosed by a surface shows the locations of the four persistent Fc-Fab2 interactions involving LC2. (**b**) Heat map in the left panel shows the bound states of four long-lived Fc-Fab2 hydrogen bonds, in three independent 750 ns MD simulations. Bound states are displayed as filled regions. (**c**) Probability densities of ${R}_{25}$, ${\theta }_{125}$, and ${\Theta }_{1256}$ computed from 600 ns simulations of 1HZH-A2G0F in the absence of the hinge and Fab1 regions. Data shown for two independent replicates. Data from two independent replicates are shown using lines of similar colors.

The HC-HC, LC-LC, and HC-LC disulfide bonds are known to stabilize the secondary structure of Abs. However, given that these bonds are symmetric across both Fab arms we looked for additional interactions that could explain the asymmetric Fab dynamics observed in our simulations. Towards this, we computed all hydrogen bonds and salt bridges across all trajectories, based on the criterion outlined in the “Materials and methods” section. Our analysis revealed the presence of 298 unique hydrogen bonds between Fab2 and Fc involving LC2 and both heavy chains HC1 and HC2, of which 38 bonds were present in over 80% of a trajectory, and of which 4 were stably bound in more than one replicate (Supplementary Figure S5, Supplementary Table 1). These four bonds involving residue pairs LC2:R211—HC1:E269 and LC2:R108–HC2:D270, in the EU numbering scheme^15^, are shown in Fig. 3(a), alongside the 16 disulfide bonds. Interestingly, we did not identify any hydrogen bonds between Fab1 and Fc which could be a potential factor that governs the differential dynamics of Fab2. The bound states of the four long-lived bonds are shown as heatmap in Fig. 3(b), where shaded and unshaded boxes denote bound and unbound states, respectively. The long-lived bonds are highly stable in two of the three replicates (E1, E2) and is either completely unbound or highly dynamic in E3 which explains the inter-replicate variations noted in Fig. 1(b-d). We also verified if the hinge regions modeled as part of homology modeling could be a potential source of asymmetric Ab dynamics. We tested this scenario in another set of simulations of 1HZH in the absence of the Fab1 region and presence of chain breaks in the hinges, see “Materials and methods” section for details. Analysis of trajectories from two replicates again showed persistent interactions between Fab2 and Fc and the corresponding statistics of fluctuations match those for the full protein, see Fig. 3(c).

### In silico protein pulling assay to disrupt Fc-Fab2 interactions

**Figure 4:**
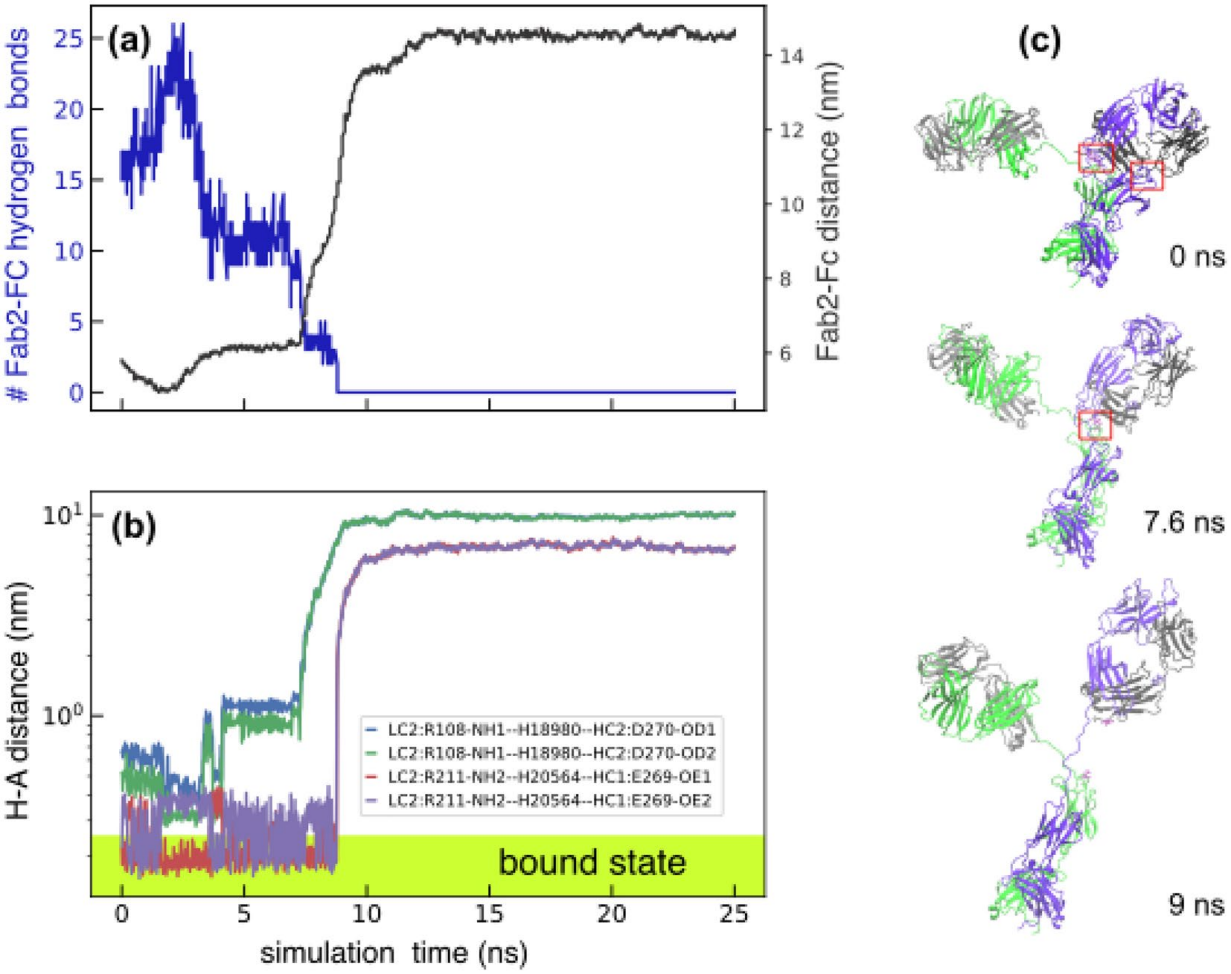
Time series of Fc-Fab2 hydrogen bonds in steered MD simulations. (**a**) Total number of Fab2-Fc hydrogen bonds (left axis) and distance between the Fc and Fab2 center of masses (right axis) for the first 25 ns. (**b**) Timeseries of the hydrogen-acceptor distance for the four long-lived hydrogen bonds showing that these bonds are completely unbound beyond 9 ns. (**c**) Ab conformations at 0, 7.6, and 9 ns. The boxes show the locations of the four long-lived hydrogen bonds identified in Fig. 3. The absence of the boxes in the “9 ns structure” indicates the loss of the hydrogen bonds.

We next performed Steered molecular dynamics simulations^16^ to disrupt the Fc-Fab2 interactions by applying an equal and opposite uniform force of 0.01 kcal/mol/nm on all atoms in the Fc and Fab2 fragments, as described in the “Materials and methods” section. It should be noted that the Steered-MD technique was not used to sample rare mAb conformations in the conventional sense. Here, it was only used as a tool to perform an in silico protein pulling assay to generate new mAb conformations with varying Fc-Fab2 distances. Figure 4(a) shows the timeseries of the total number of hydrogen bonds between Fc and Fab2, and the distance between their center of masses (also see supplementary Figure S6). Despite the applied external force, the separation between the Fc and Fab2 is stable up to ~ 9 ns. However, the hydrogen bonds between Fc-Fab2 are reorganized in response to the applied force and steadily decrease during this interval until all bonds are broken at ~ 9 ns. At this junction, the constrained Fab2 arm is released, and the Ab conformation adopts a symmetric-**Y** shape as shown in Fig. 4(c). Fluctuations in the hydrogen-acceptor distance for the four long-lived bonds identified earlier are shown in Fig. 4(b). Both hydrogen bonds involving LC2:R211—HC1:E269 remain bound until 9 ns while the other two involving LC2:R108—HC2:D270 instantly unbind upon application of the force, also see Supplementary Figure S6. This suggests that LC2:R211—HC1:E269 mediated binding is the most relevant interaction for the reaction coordinate used in our model. Taken together our analysis indicates that Fc-Fab2 interactions via hydrogen bonding can stabilize non-symmetric Fab conformations which in turn can alter Ab function and binding. We computed the total external work done by all atoms subjected to the pulling force as $W= {\sum }_{i}{ f}_{Fab2} {\overrightarrow{{\varvec{R}}}}_{{\varvec{i}}}\cdot \overrightarrow{{\varvec{r}}}-{\sum }_{j}{ f}_{FC} {\overrightarrow{{\varvec{R}}}}_{{\varvec{j}}}\cdot \overrightarrow{{\varvec{r}}},$ with ${\overrightarrow{{\varvec{R}}}}_{{\varvec{i}}}$ being the displacement of atom $i$ in the “9 ns structure” with respect to the “0 ns structure”. $\overrightarrow{{\varvec{r}}}$ denotes the vector connecting the center of masses of the Fc and Fab2 regions in the “0 ns structure” as described in the “Materials and methods” section. We estimated the total work to transform the “0 ns structure” to the “9 ns structure” to be $W=1155.6\mathrm{kcal}/\mathrm{mol}$, which following Jarzynski equality theorem is the maximum possible value of all changes in the internal energy of the molecule, that also includes the contributions from Fc-Fab2 interactions^17^.

### Fab arms display identical dynamics in the absence of Fc-Fab interactions

**Figure 5:**
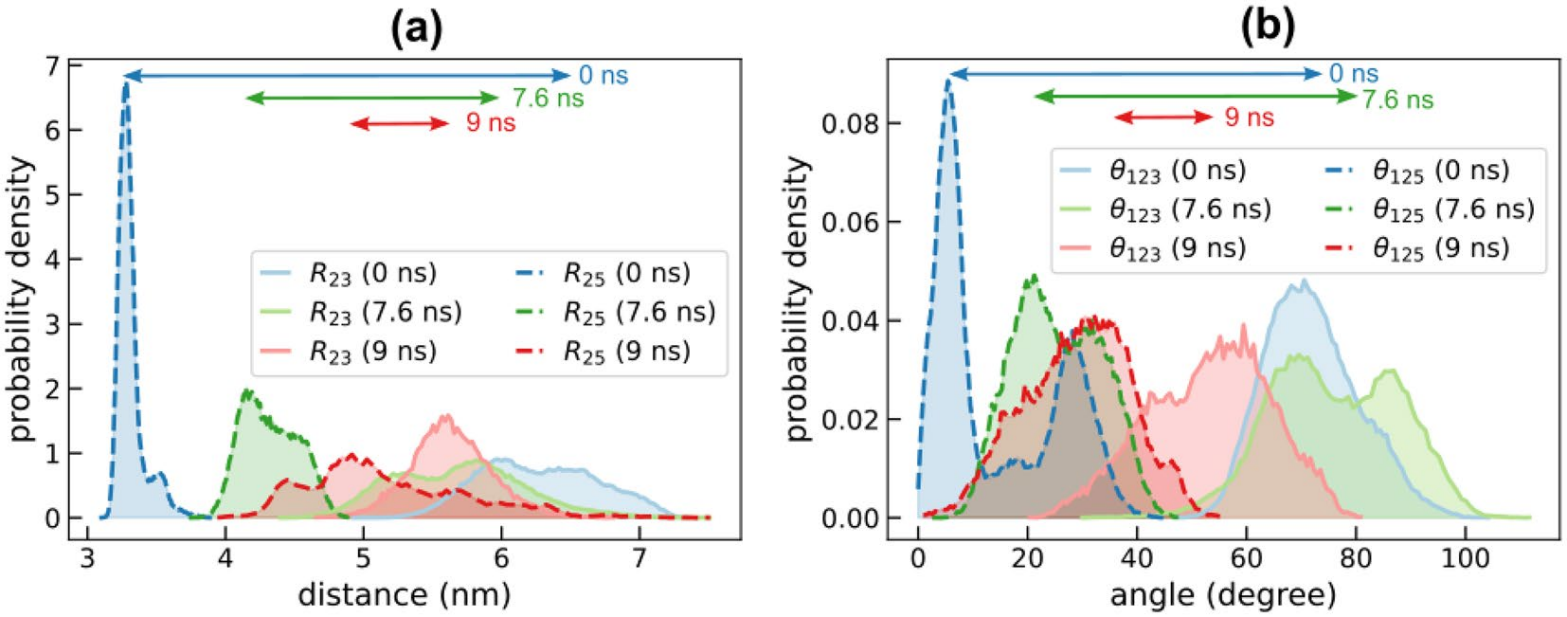
Comparison of Fab-Fc distance and orientation fluctuations in 200 ns MD simulations using three different structures extracted at 0, 7.6, and 9 ns of the steered MD simulations discussed in Fig. 4. (**a**) Panel shows the probability distribution of ${R}_{23}$ and ${R}_{25}$ for the three sets of simulations. (**b**) Panel shows the probability distribution of ${\theta }_{123}$ and ${\theta }_{125}$. The distributions were computed from 3 replicates (0 ns) and two replicates for the rest. The length of the double-headed arrows displayed on the top of each panel is proportional to the relative separation between the Fab1 and Fab2 peaks.

We performed additional 200 ns NPT simulations of this antibody starting from the molecular structures extracted at 0, 7.6 and 9 ns of the steered MD simulations, shown in Fig. 4(c). The probability distributions of Fab-Fc distance and orientational fluctuations are shown in Fig. 4(a) and (b), and the corresponding timeseries are shown in Supplementary Figures S7 and S8. Fab-Fc distances ${R}_{23}$ and ${R}_{25}$, corresponding to Fab1 and Fab2, respectively, show significant overlap in simulations of the “9 ns structure” compared to relative separations observed for the “7.6 ns” and “0 ns” structures. A similar trend is also seen for the orientational fluctuations ${\theta }_{123}$ and ${\theta }_{125}$. It should be noted that the primary difference between the “0 ns” and “9 ns” structures is the presence of 38 long-lived Fc-Fab2 hydrogen bond interactions in the former, and the complete loss of these interactions in the latter. These results further cement our observation that non-identical Fc-Fab interactions lead to non-identical Fab dynamics, described earlier in Fig. 1. Additionally, loss of Fc-Fab2 interactions not only alters Fab2 dynamics but also subtly modulates the equilibrium states of Fab1 as shown by the shift in Fab1 probability density curves in Fig. 5. The observed shift could be a potential signature of allosteric interactions between Fab1 and the FC and/or Fab2. While allosteric coupling between Fab and Fc have been reported earlier, to the best of our knowledge this study presents first evidence for such coupling between the Fabs.

## Discussions

We have studied the internal dynamics of human IgG structure, 1HZH, using long all atom molecular dynamics simulations and show that Fab domains exhibit non-identical dynamics. We have identified strong, long-lived, non-covalent interactions between Fc and Fab that severely constrain the translational and rotational dynamics of Fab. We have also shown using steered MD simulations that the Fc-Fab hydrogen bonds, involving LC and both heavy chains, are very stable up to a threshold applied force. Beyond this threshold the Fc-Fab interactions are completely lost and the Ab settles in a symmetric state where both Fab domains exhibit identical dynamics and show symmetric **Y** conformations. More importantly, Fab-Fc interactions subtly modulate the dynamics of the other Fab as shown in Fig. 5. These findings also demonstrate complex allosteric interactions that govern Ab dynamics.

The Fc and Fab regions were conventionally thought of as independent domains, each with a specific role. Several recent findings have challenged this notion, see Janda et al.^18^ for an extensive review on this topic. Notably, it has known for long that antigen binding to Fab induces structural changes in the Fc^18,19^. It was recently shown that antigen-Fab binding mediated allosteric interactions increased the affinity of IgG1 Fc regions for Fc$\gamma$n receptors^20^. Another study by Zhao et al.^21^ demonstrated conformational changes in both the glycans and Fc in response to Fab-antigen binding. It has also been shown in multiple studies that orientations of the V_H_–V_L_ and the C_H_1–CL domains alters the state of the paratope and as a result modulates the affinity for Fab-antigen binding^12,13,22^. In this context, variations in the Ab dynamics affect structure which in turn modulates function. The role of mAb dynamics on mAb binding was demonstrated in a coarse grained molecular dynamics study by De Michele and coworkers^23^ where they showed that the “Fab internal flexibility is key to maximizing bivalent binding”.

Although the results presented in this work are specific to one IgG1 structure, our analysis provides useful insights into the role of Fc-Fab interactions in governing Ab internal dynamics. These findings naturally evoke the question “what is the role of Ab conformer states in its biological activity and do Abs show conformer state dependent antigen-binding affinity?” We plan to address these aspects in upcoming works. Finally, it should also be noted that the population of conformers with Fc-Fab interactions are not well characterized at this point and it is imperative for future works to subclassify immunoglobulins based on their shape asymmetries.

## Materials and methods

### Preparation of 1HZH structure

We obtained the human IgG1 crystal structure (1HZH) from RSCB protein data bank. We used Discovery Studio 2020 from BIOVIA to clean up the protein and used homology modeling to fix the missing hinge regions. The resulting structure was equilibrated for 10 ns. We prepared N-glycosylated forms of 1HZH by attaching A2G0F glycan^24^ to residue N297 in each of the heavy chains HC1 and HC2 and the resulting structure files were used as inputs in our simulations. We use the EU numbering scheme^15^ to number all residues. The modeled structure contained 16 disulfide bonds that were retained in all our simulations.

### Molecular dynamics simulations

We performed all molecular dynamics simulations presented in this article with the open source tool OpenMM^25^ using CHARMM36 force field for proteins (CHARMM36m^26^), carbohydrates and glycopeptides. All simulations were run in an implicit solvent following the Hawkins-Cramer-Truhlar GBSA model^27^, with salt concentration of 150 mM, and a timestep of 2 fs. In all our simulations the initial structure was first minimized, then equilibrated for 1 ns, followed by production runs. We performed all simulations at 300 K with a friction coupling of 1 ps^−1^. Typical runs of a N-glycosylated 1HZH, with 20,984 atoms, on a single NVIDIA GTX 1080 GPU card yielded 10–12 ns a day. We analyzed all simulation trajectories using MDTraj^28^. We employed the MDTraj implementation of Baker-Hubbard method to compute the hydrogen bonds between residues. In our calculations, we used the following cutoffs to determine bound state: hydrogen-acceptor distance < 2.5 Å and donor-hydrogen-acceptor angle > 120°. For explicit solvent simulations, the glycosylated protein was solvated in a TIP3P cubic water box with 15 Å padding and the system was neutralized with 150 mM NaCl counterions. Long-range non-bonded electrostatic interactions were handled using Particle-Ewald-Mesh, with a cutoff of 0.8 nm. All hydrogen bonds were constrained allowing us to use a timestep of 2 fs.

### Principal component analysis

We studied the essential dynamics of 1HZH by performing a principal component analysis of the ${C}_{\alpha }$ atoms. We first aligned every frame in the trajectory to the Fc domain of the first frame, following which we constructed and solved the covariance matrix using the PCA module in the python scikit-learn library. We next projected the positions of the ${C}_{\alpha }$ atoms on to each principal vector and evaluated the contributions of each atom to the essential dynamics.

### Steered MD simulations

We performed steered MD simulations by applying constant forces ${-f}_{FC }\overrightarrow{{\varvec{r}}}$ and ${f}_{Fab2 }\overrightarrow{{\varvec{r}}}$ on all atoms in the Fc and Fab2 regions, respectively. Here, $\overrightarrow{{\varvec{r}}}$ denotes the initial vector connecting the center of masses of Fc and Fab2. In our study, we chose ${f}_{FC}={f}_{Fab2}=0.01 \mathrm{kcal}/\mathrm{mol}/\mathrm{nm}$, a choice that ensured that the pulled structures are in quasi-equilibrium.

### Preparation of 1HZH structure without Fab1 and hinge break

To examine the role of the hinge regions in mediating Fc-Fab2 interactions, we performed additional simulations of 1HZH in the absence of its Fab1 region alongside chain breaks in the hinge regions. Towards this we deleted residues HC1:Q1-H224, LC1:E1-C214, and introduced a chain break between residues H224 and T225 in both heavy chains, using Discovery Studio 2020 from BIOVIA. The resulting structure containing 14,186 atoms were prepared for MD simulations as described for 1HZH.

## Supplementary Information

Supplementary Information 1.

Supplementary Information 2.
