# Supplementary material for: Non-covalent Fc-Fab interactions significantly alter internal dynamics of an IgG1 antibody
Source: Sci Rep. 2022 Jun 4;12:9321. doi: 10.1038/s41598-022-13370-3 (PMC9167292; doi:10.1038/s41598-022-13370-3)
Supplement: Supplementary file 1 — Supplementary Information 1. [file 41598_2022_13370_MOESM1_ESM.pdf]

Supplementary Information for “Non-covalent Fc-Fab interactions significantly alter internal dynamics of an IgG1 antibody”

Ramakrishnan Natesan, Neeraj J. Agrawal
Amgen Inc., Process Development, Cambridge, Massachusetts 02141

Amgen Proprietary - Confidential

Internal Use Only General Business

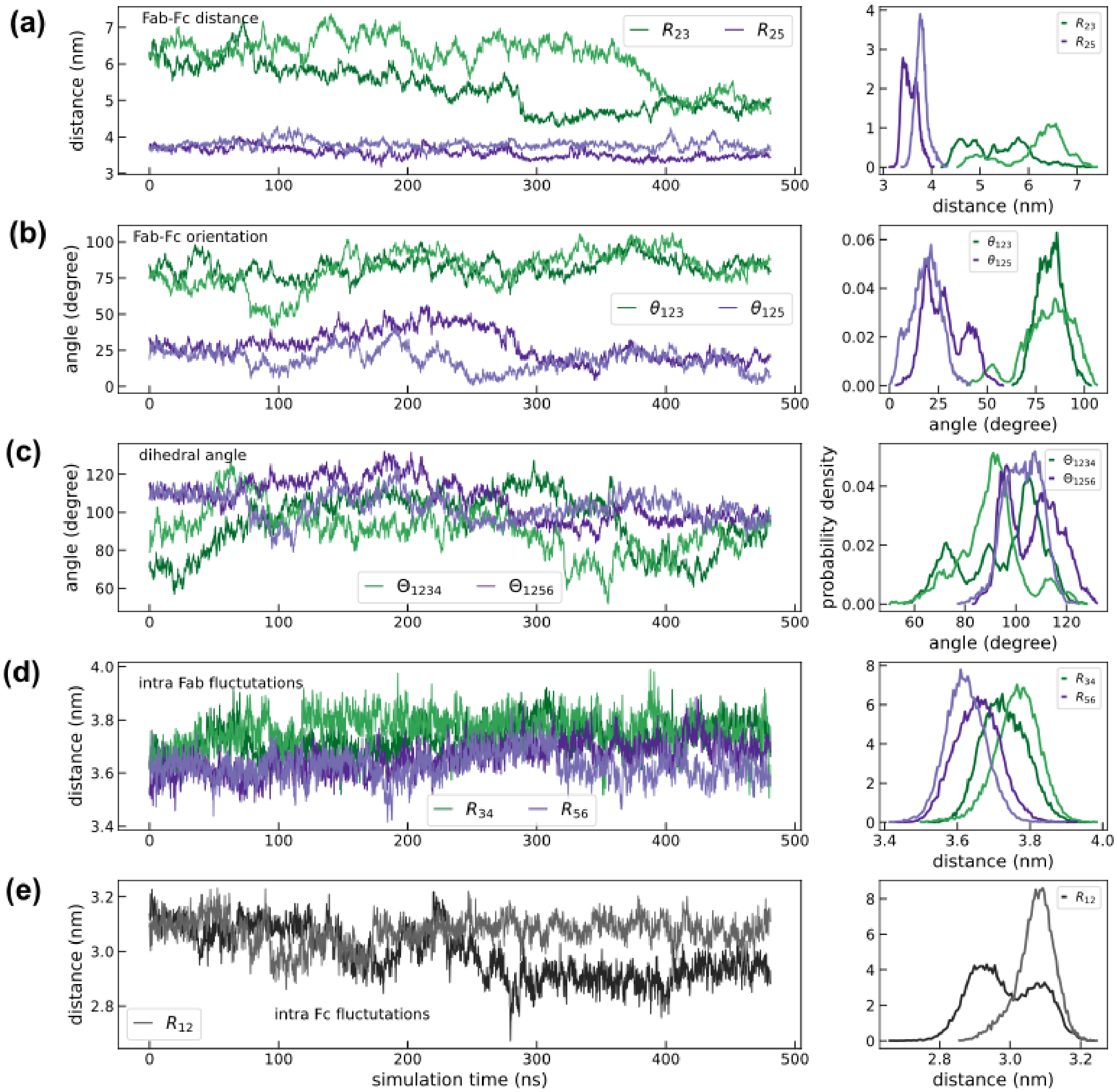

Figure S1: Statistics of Fab and Fc fluctuations from two independent 500 ns NPT explicit solvent simulations of glycosylated-1HZH. Shown are **(a)** Fab-Fc displacements R_{23} and R_{25}, **(b)** Fab-Fc orientation θ_{234} and θ_{256}, **(c)** Fc-Fab dihedral angles Θ_{1234} and Θ_{1256}, **(d)** intra Fab displacements R_{34} and R_{56}, and **(e)** intra-Fc displacement R_{12}. Data from three independent replicates are shown using lines of similar colors.

Amgen Proprietary - Confidential

Internal Use Only General Business

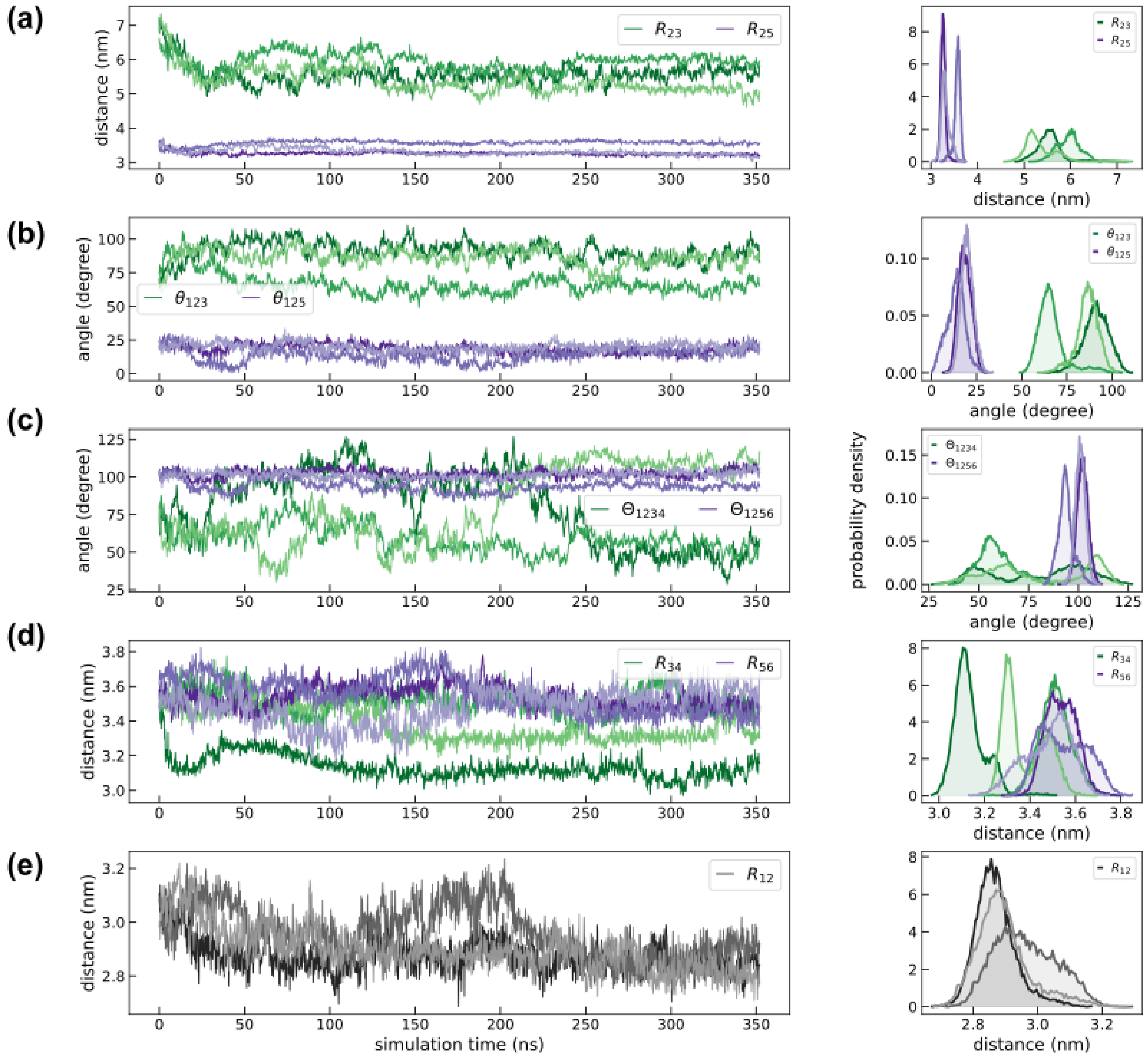

Figure S2: Statistics of Fab and Fc fluctuations from three independent 350 ns NPT implicit solvent simulations of non-glycosylated 1HZH. Shown are **(a)** Fab-Fc displacements R_{23} and R_{25}, **(b)** Fab-Fc orientation θ_{234} and θ_{256}, **(c)** Fc-Fab dihedral angles Θ_{1234} and Θ_{1256}, **(d)** intra Fab displacements R_{34} and R_{56}, and **(e)** intra-Fc displacement R_{12}. Data from three independent replicates are shown using lines of similar colors.

Amgen Proprietary - Confidential

Internal Use Only General Business

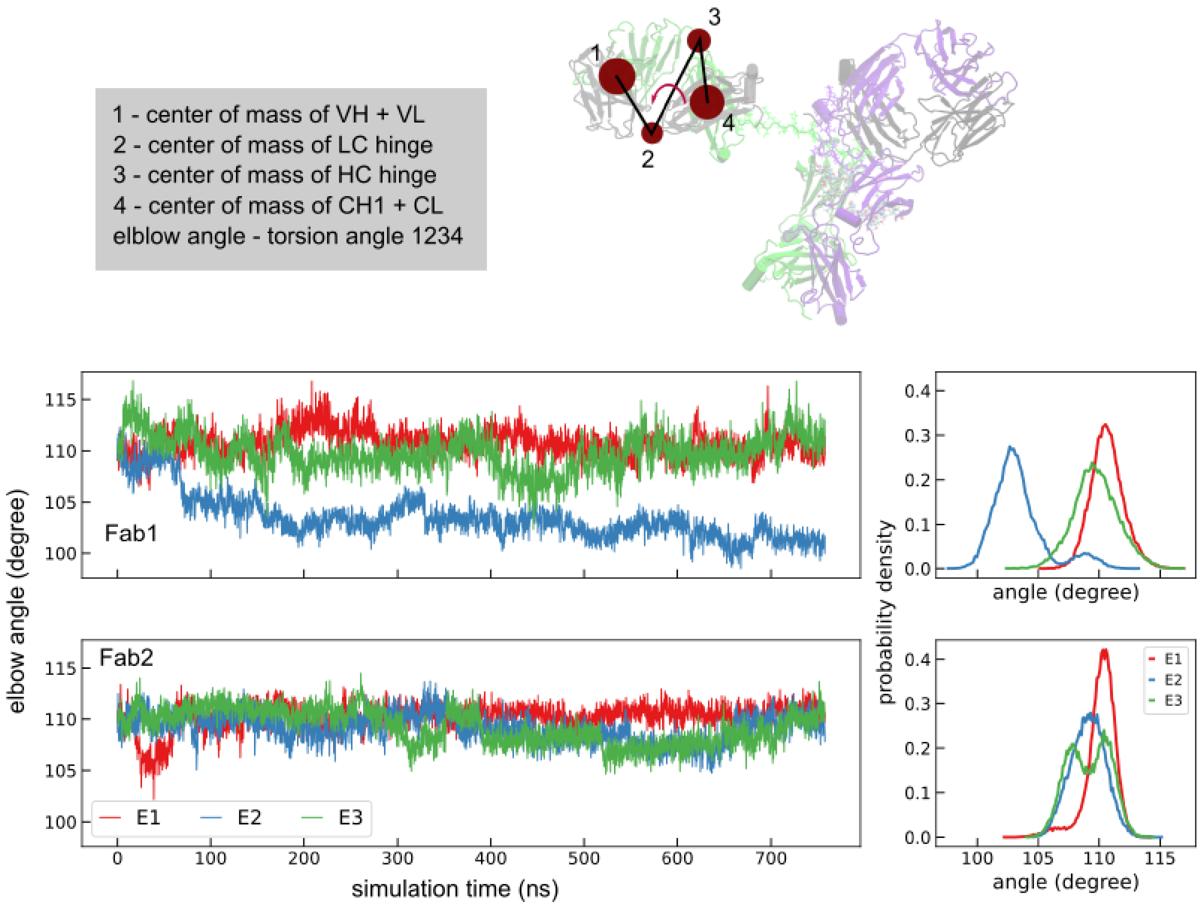

Figure S3: (Top) Illustration of elbow angle calculation for Fab1 following reference [1]. The elbow angle was computed as the torsion angle connecting the center of masses of (1) VH+VL, (2) hinge linking VL to CL, (3) hinge linking VH to CH1 and (4) CL+CH1. (Bottom) Time series of elbow angles from 750 ns glycosylated-1HZH implicit solvent simulations shows that Fab1 and Fab2 display nearly identical elbow angle dynamics.

Amgen Proprietary - Confidential

Internal Use Only General Business

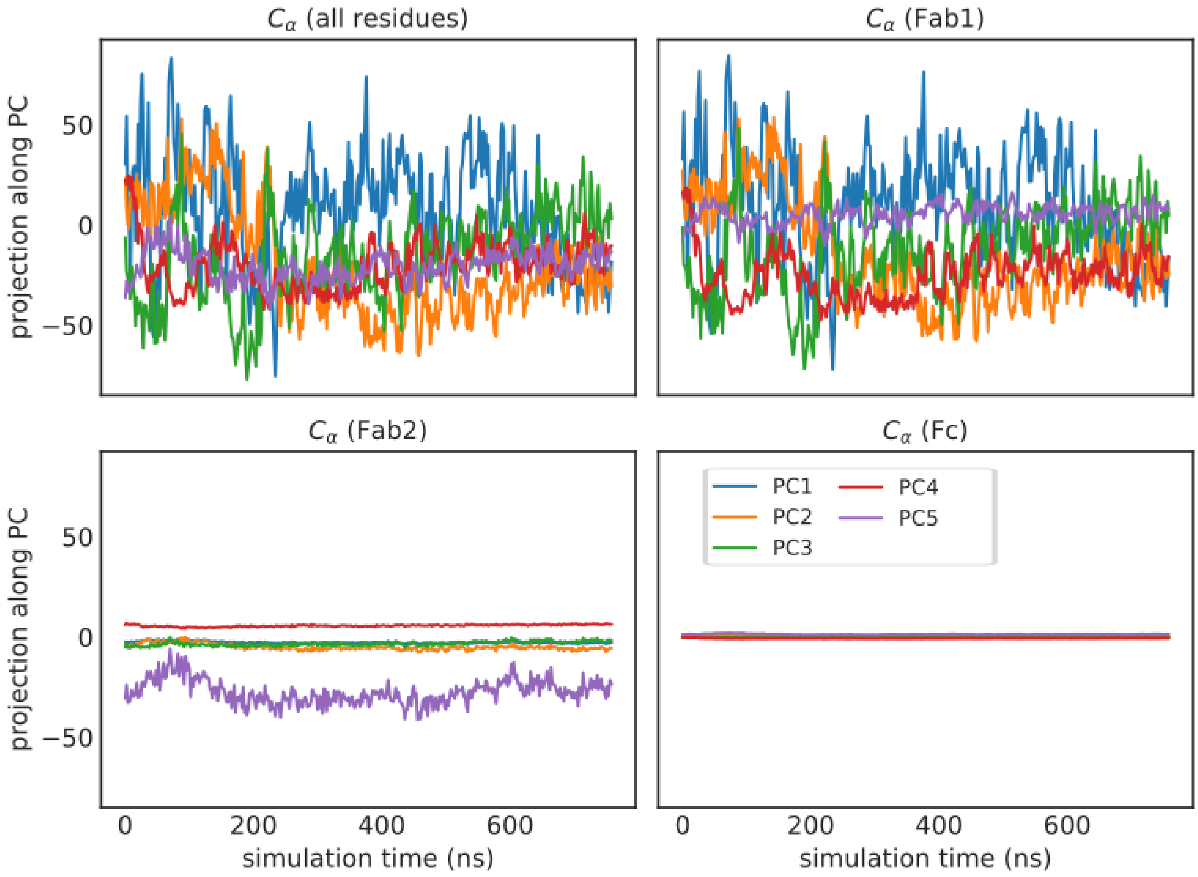

Figure S4: Principal component analysis of 750 ns glycosylated 1HZH dynamics in implicit solvent. Time series showing the contributions to the first five principal vectors (PC1-PC5) from C_α atoms in all residues (top left), Fab1 arm (top right), Fab2 arm (bottom left), and Fc domain (bottom right). Fab1 motion dominates the essential dynamics since the contributions from all residues closely mirror that of Fab1. Contributions from Fab2 and Fc domains to the first four principal components are negligible.

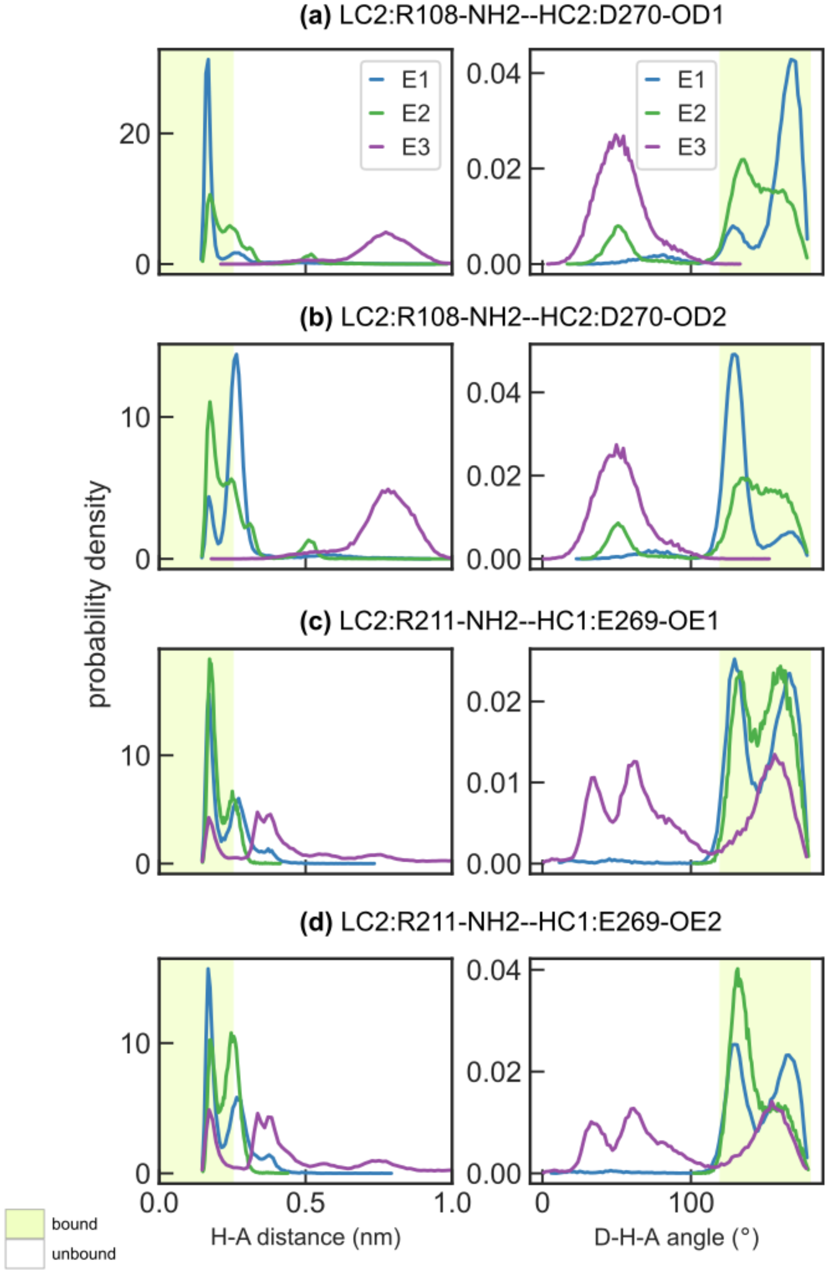

Figure S5: (a-d) Left and right panels show the probability distribution of the hydrogen-acceptor (H-A) distance and donor-hydrogen-acceptor (D-H-A) angle, respectively, for the four long-lived hydrogen bonds identified in Main Figure 2. Data from three independent replicates (E1-E3) 750 ns NPT simulations of N-glycosylated 1HZH are shown in different colors. The shaded region in each panel correspond to bound states while unshaded regions represent unbound states. Following the documentation for MDTraj [2], we set H-A and D-H-A cutoff for bound state as < 0.25 nm and >120°, respectively.

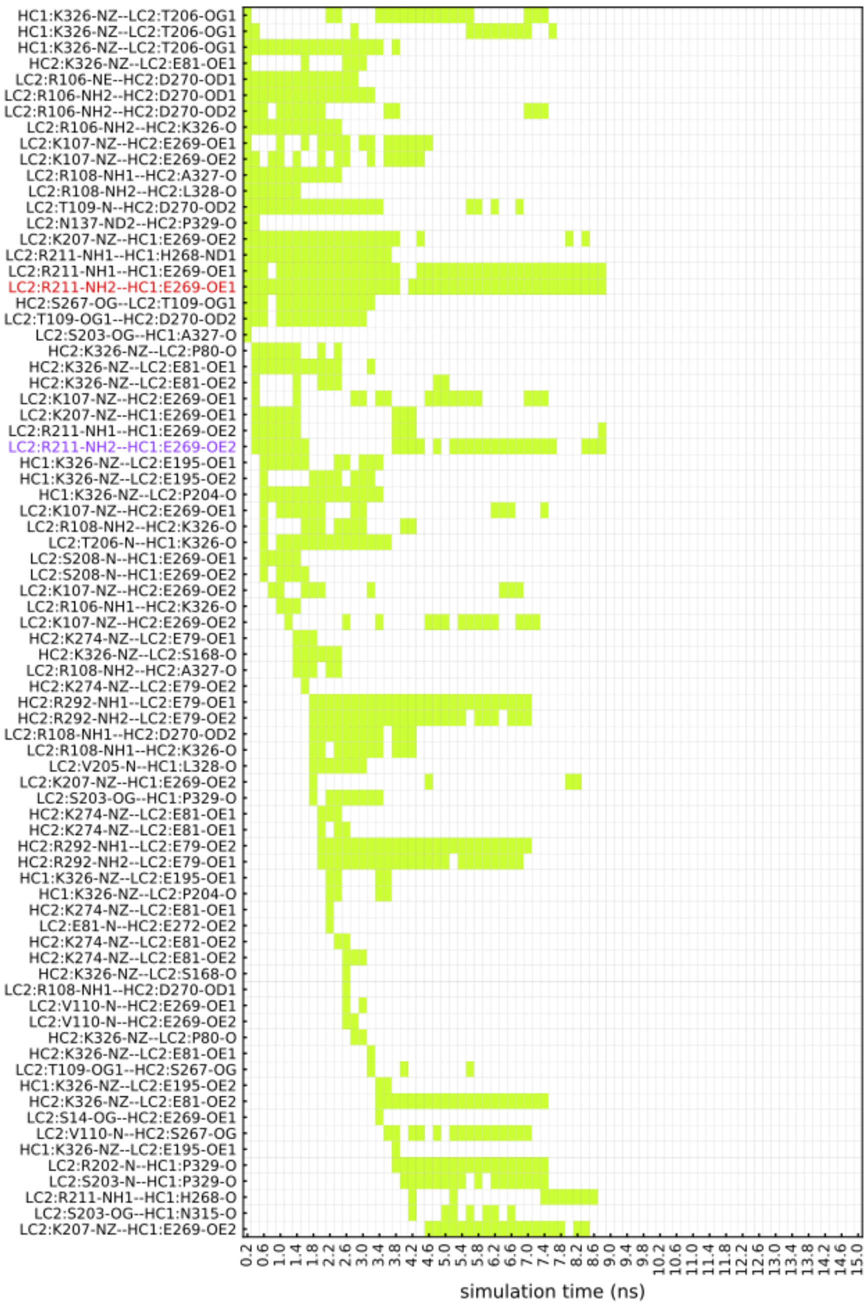

Figure S6: Heatmap shows the timeseries of Fc-Fab2 hydrogen bonds in Steered MD simulations of N-glycosylated 1HZH. The bonds are named per the convention DONOR--HYDROGEN--ACCEPTOR. Only two the four long-lived bonds, identified in Figure 2, persist until 9 ns and the corresponding labels are colored differently.

Amgen Proprietary - Confidential

Internal Use Only General Business

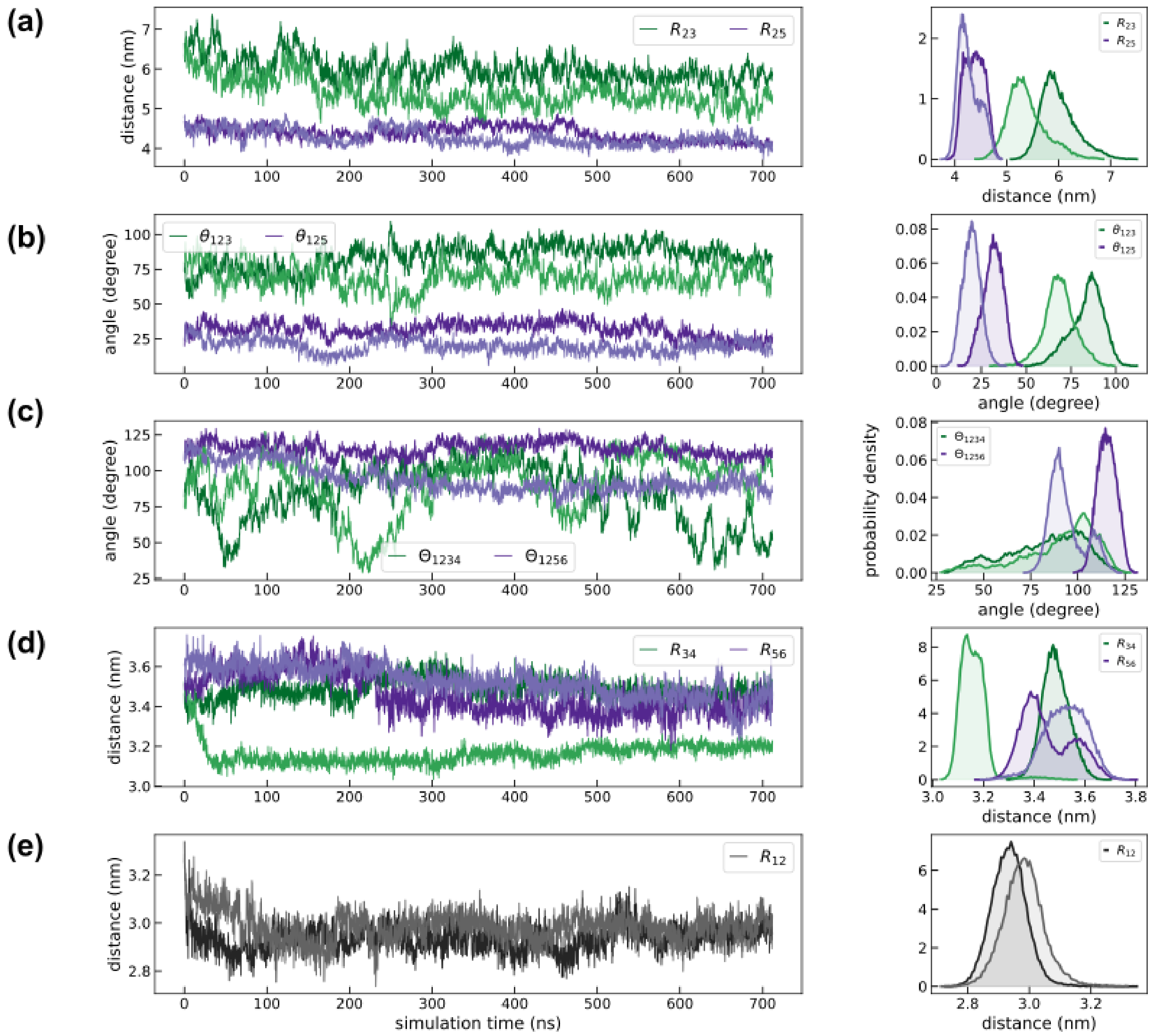

Figure S7: Statistics of distance and orientational fluctuations for NPT simulations starting from the 7.6 ns structure with some degree of Fc-Fab2 interactions. Shown are **(a)** Fab-Fc displacements R_{23} and R_{25}, **(b)** Fab-Fc orientation θ_{234} and θ_{256}, **(c)** Fc-Fab dihedral angles Θ_{1234} and Θ_{1256}, **(d)** intra Fab displacements R_{34} and R_{56}, and **(e)** intra-Fc displacement R_{12}. Data from three independent replicates are shown using lines of similar colors. Fab2 shows slightly non-identical dynamics compared to Fab1.

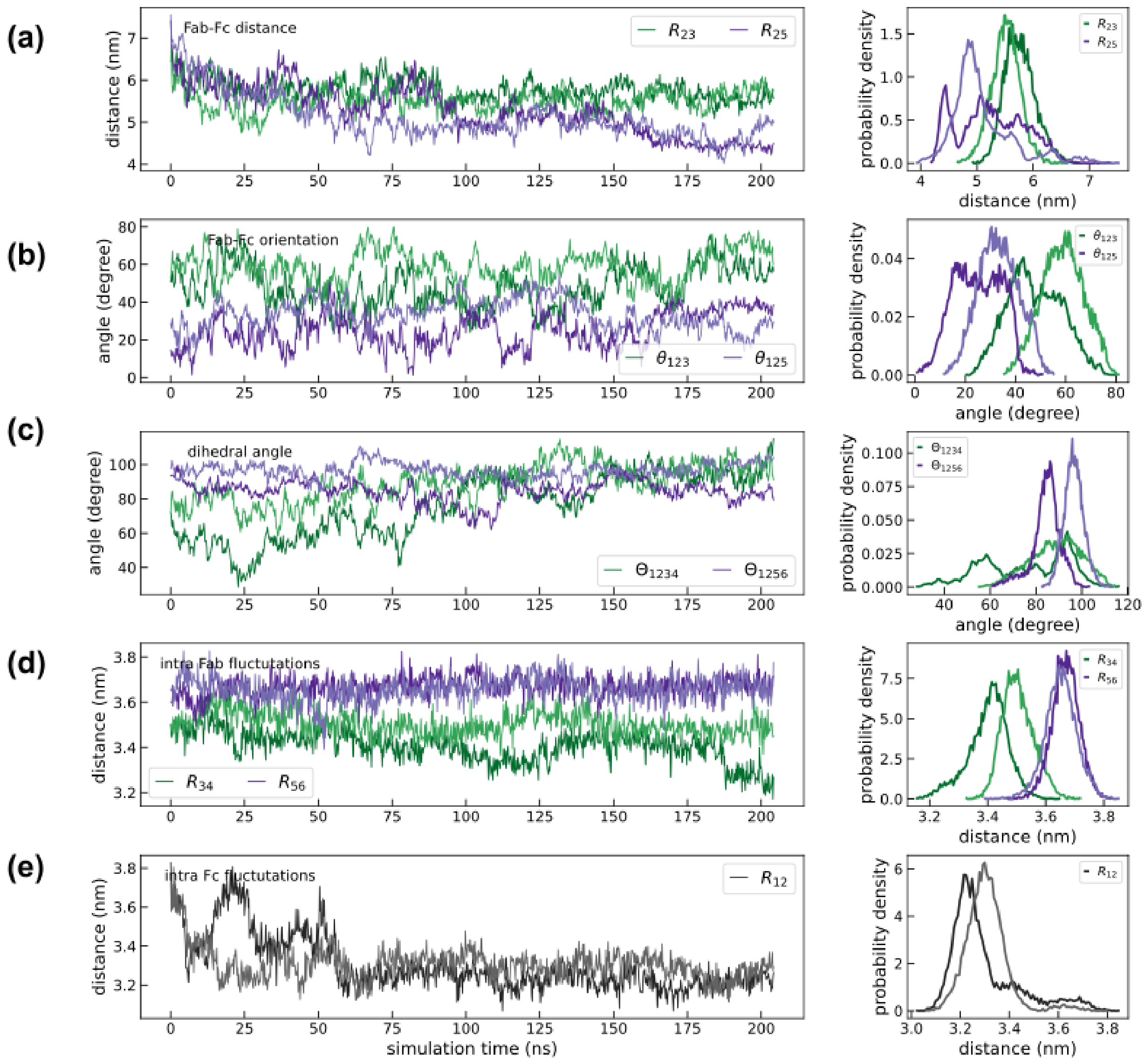

Figure S8: Statistics of distance and orientational fluctuations for 200 ns NPT simulations starting from the "9 ns structure" that lacks any Fc-Fab2 interactions. Shown are **(a)** Fab-Fc displacements R_{23} and R_{25}, **(b)** Fab-Fc orientation θ_{234} and θ_{256}, **(c)** Fc-Fab dihedral angles Θ_{1234} and Θ_{1256}, **(d)** intra Fab displacements R_{34} and R_{56}, and **(e)** intra-Fc displacement R_{12}. Data from three independent replicates are shown using lines of similar colors. Both Fab arms display identical dynamics in the absence of any Fc-Fab2 interactions.

Amgen Proprietary - Confidential

Internal Use Only General Business

References

1. Fernandez-Quintero, M.L., et al., *Surprisingly Fast Interface and Elbow Angle Dynamics of Antigen-Binding Fragments.* Front Mol Biosci, 2020. **7**: p. 609088.
2. McGibbon, R.T., et al., *MDTraj: A Modern Open Library for the Analysis of Molecular Dynamics Trajectories.* Biophys J, 2015. **109**(8): p. 1528-32.

Amgen Proprietary - Confidential

Internal Use Only General Business
